# A systematic review: comparative analysis of the effects of propofol and sevoflurane on postoperative cognitive function in elderly patients with lung cancer

**DOI:** 10.1186/s12885-019-6426-2

**Published:** 2019-12-23

**Authors:** Haitao Sun, Guohua Zhang, Bolun Ai, Huimin Zhang, Xiangyi Kong, Wan-Ting Lee, Hui Zheng, Tao Yan, Li Sun

**Affiliations:** 1 Department of Anesthesiology, National Cancer Center/National Clinical Research Center for Cancer/Cancer Hospital, Chinese Academy of Medical Sciences and Peking Union Medical College, Beijing, 100021 China; 2 Department of Breast Surgical Oncology, National Cancer Center/National Clinical Research Center for Cancer/Cancer Hospital, Chinese Academy of Medical Sciences and Peking Union Medical College, Beijing, 100021 China; 3 Department of Gastroenterology, Inner Mongolia’s Peoples’ Hospital, Inner Mongolia, 010000 China; 4 Mater Hospital Brisbane Queensland Medical Program, The University of Queensland, Brisbane, Australia; 5 Department of Anesthesiology, National Cancer Center/National Clinical Research Center for Cancer/Cancer Hospital & Shenzhen Hospital, Chinese Academy of Medical Sciences and Peking Union Medical College, Shenzhen, 518116 China

**Keywords:** Propofol, Sevoflurane, Cognitive function, Lung Cancer, Meta-analysis

## Abstract

**Background:**

The potential risk for cognitive impairment following surgery and anesthesia is a common concern, especially in the elderly and more fragile patients. The risk for various neurocognitive effects is thus an area of importance. The independent impact of surgery and anesthesia is still not known. Likewise, the independent effect of different drugs used during anesthesia is a matter of debate, as is the number and amounts of drugs used and the “depth of anesthesia.” So, understanding the drug-related phenomenon and mechanisms for postoperative cognitive impairment is essential. This meta-analysis aims to compare the effects of propofol and sevoflurane anesthesia on postoperative cognitive function in elderly patients with lung cancer.

**Methods:**

This study is a systematic review and meta-analysis for controlled clinical studies. Public-available online databases were searched to identify eligible randomized placebo-controlled trials or prospective cohort studies concerning the effects of propofol and sevoflurane on postoperative cognitive function. The primary endpoints are postoperative mini-mental state examination (MMSE) scores at various time points; the secondary endpoint is the serum S100beta concentration 24 h after surgery. Standard mean differences (SMDs) along with 95% confidence intervals (CIs) were extracted and analyzed using random or fixed-effects models. Analyses regarding heterogeneity, risk of bias assessment, and sensitivity were performed.

**Results:**

We searched 1626 eligible publications and 14 studies of 1404 patients were included in the final analysis. The majority of included studies had been undertaken in Asian populations. Results suggested that propofol has a greater adverse effect on cognitive function in the elderly patients with lung cancer than sevoflurane. There were significant differences in issues of MMSE 6 h (11 studies; SMD -1.391, 95% CI -2.024, − 0.757; *p* < 0.001), MMSE 24 h (14 studies; SMD -1.106, 95% CI -1.588, − 0.624; *p* < 0.001), MMSE 3d (11 studies; SMD -1.065, 95% CI -1.564, − 0.566; *p* < 0.001), MMSE 7d (10 studies; SMD -0.422, 95% CI -0.549, − 0.295; *p* < 0.001), and serum S100beta concentration at 1 day after surgery (13 studies; SMD 0.746, 95% CI 0.475, 1.017; *p* < 0.001).

**Conclusion:**

Propofol has a more significant adverse effect on postoperative cognitive function in elderly patients with lung cancer than sevoflurane.

## Background

Cognitive impairment is a neurological disorder that occurs in adults, which involves cognitive disorders with impairment in instrumental activities of daily living [1, 2]. Previous studies have shown that about 234 million patients worldwide undergo surgery each year, and about 41% of elderly patients have cognitive impairment after surgery or anesthesia. Thirteen percent of patients still have cognitive impairment 3 months after discharge [3, 4]. Cognitive impairment severely affects the prognosis of patients who have undergone general anesthesia surgeries, especially elderly patients, including decreased quality of life, loss of independence, and increased mortality [5]. Patients with lung cancer frequently encounter postoperative cognitive dysfunction. In elderly patients, severe cognitive impairment is more likely to occur after anesthesia. This may be due to a combination of multiple factors, such as inflammation caused by surgical trauma, infection, opioids, stress, and sleep disorders [6]. Because the incidence of cognitive impairment is positively correlated with the duration of anesthesia, general anesthetic drugs are thought to be one of the causes of cognitive impairment in elderly patients [7].

Worldwide, lung cancer occurred in approximately 1.8 million patients in 2012 and caused an estimated 1.6 million deaths [8]. This is increased from 1.6 million new diagnoses and 1.4 million lung cancer deaths in 2008 [9]. The treatment for early-stage lung cancer is mainly surgical treatment. Propofol and sevoflurane are the most commonly used general anesthetic drugs in clinical practice [10]. However, in terms of causing postoperative cognitive functions, there are still controversies regarding the use of propofol anesthesia or sevoflurane anesthesia in elderly patients with lung cancer. Current published studies on this topic were with relatively smaller sample sizes and were lack of consistency. Therefore, we conducted this systematic review and meta-analysis to derive a pooled estimate of the effects of propofol and sevoflurane on postoperative cognitive functions in patients with lung cancer, with an improved statistical power as compared to individual studies. The primary endpoints are postoperative mini-mental state examination (MMSE) scores at various time points; the secondary endpoints are serum S100beta concentration 24 h after surgery. S100β is glial-specific and is expressed primarily by astrocytes. It functions in the process of neurite extension, proliferation of melanoma cells, stimulation of Ca2+ fluxes, inhibition of PKC-mediated phosphorylation, astrocytosis, axonal proliferation, and inhibition of microtubule assembly. In the developing central nervous system (CNS), it acts as a neurotrophic factor and neuronal survival protein. In the adult organism, it is usually elevated due to nervous system damage, which makes it a potential clinical marker. S100β is involved in the regulation of cell shape, cell growth, energy metabolism, cell-to-cell communication, contraction, and intracellular signal transduction. The correlation between serum S100β protein levels and cognitive dysfunction.

## Methods

### Research protocol overview

Public-available online databases were searched to identify eligible randomized placebo-controlled trials or prospective cohort studies concerning the effects of propofol and sevoflurane on postoperative cognitive function. The primary endpoints are postoperative mini-mental state examination (MMSE) scores at various time points; the secondary endpoint is the serum S100beta concentration 24 h after surgery. Standard mean differences (SMDs) along with 95% confidence intervals (CIs) were extracted and analyzed using random or fixed-effects models. Analyses regarding heterogeneity, risk of bias assessment, and sensitivity were performed.

### Search strategies

We searched the databases of Embase, Pubmed, The Cochrane Library, Web of Science, and China National Knowledge Infrastructure (CNKI). Retrieval time is from the database construction time to March 2018. The English search words include propofol, sevoflurane, cognitive, and lung cancer. The Chinese search words are the Chinese translation of the above words. There are no language and time limits. For the retrieved documents, we further tracked their references to include the ones that met the inclusion criteria.

### Inclusion and exclusion criteria

This study analyzed studies with a population of > 60 years-old, ASA class I to III patients who had scheduled for lung cancer surgeries, and received propofol or sevoflurane during anesthesia. The primary endpoints are postoperative mini-mental state examination (MMSE) scores at various time points; the secondary endpoint is serum S100beta concentration 24 h after surgery. Specific inclusion criteria and exclusion criteria were reported in Table 1.

**Table 1:** Inclusion criteria for study selection in this meta-analysis

Number	Inclusion criteria
1	Original prospective cohort studies or randomized controlled trials (RCTs) published in full text and those for which we had full access to all original data and protocols.
2	The studies evaluated the differences of the effect of propofol and sevoflurane on postoperative cognitive functions.
3	Regarding the intervening measures between different groups, the only difference is that the two groups received propofol or sevoflurane, respectively. Other conditions should be the same.
4	Human studies.
5	Predefined outcomes: incidence of postoperative MMSE scores and the plasma protein S100β at various time points.
6	No minimal sample size or dosing regimen was required for inclusion.
Number	Exclusion criteria
1	The study did not have a control group of patients without propofol use or sevoflurane use.
2	They were case studies or case series.
3	The report focused exclusively on other topics or outcomes.
4	No human data were included.
5	Except for the difference of anaesthetic administration, there were other differences between the experimental groups and the control groups.
6	Reviews and duplicated publications.

### Data extraction and quality evaluation

Two researchers independently conducted literature screening and quality evaluation of the obtained documents. In case of disagreement, they would reach an agreement by discussion or invite a third party to adjudicate. Data extraction: 1) necessary information included in the study, such as first author and publication date; 2) general data of patients in the experimental group and control group, such as the sex, age, America Society of Anesthesiologist (ASA) classification, intervention measures, operations, etc.; 3) anesthesia methods, including methods of inducing anesthesia and maintenance of anesthesia, and the drug doses; 4) outcomes, including preoperative and postoperative MMSE scores, changes in serum S100beta concentration before and after surgery. The main characteristics of the included studies were summarized in Table 2.

**Table 2:** Characteristics of studies included in the meta-analysis

Author	Year	Country	Sex (M/F)	Age	Surgery	ASA grade	Outcomes	Propofol group		Sevoflurane group	
								Method	No.	Method	No.
Yu et al.	2012	China	44 /36	68.8 ± 3.8	Lung cancer operation	I~II	①②③④⑤⑥	Induction: midazolam, fentanyl, rocuronium, etomidate; Maintain: propofol	40	Induction: midazolam, fentanyl, rocuronium, etomidate; Maintain: sevoflurane	40
Tang et al.	2014	China	38 /32	70.0 ± 11.7	Lung cancer operation	I~II	①②③④⑤⑥	Induction: etomidate, midazolam, fentanyl, rocuronium; Maintain: propofol	35	Induction: etomidate, midazolam, fentanyl, rocuronium; Maintain: sevoflurane	35
Sun et al.	2014	China	77 /29	72.2 ± 2.6	Lung cancer operation	N	①②③④⑤⑥	Induction: fentanyl and vecuronium bromide; Maintain: propofol 2~4 mg/kg/min	53	Induction: fentanyl and vecuronium bromide; Maintain: sevoflurane	53
Cui et al.	2015	China	94 /76	69 ± 12.9	Lung cancer operation	N	①③⑤⑥	Induction: fentanyl, etomidate, vecuronium bromide; Maintain: propofol 2~4 mg/kg/min	80	Induction: fentanyl, etomidate, vecuronium bromide; Maintain: sevoflurane 1%~ 3%	80
Zhang et al.	2016	China	101 /91	60.0 ± 6.4	Lung cancer operation	N	①③④⑥	Induction: midazolam, fentanyl, rocuronium, etomidate; Maintain: propofol	96	Induction: midazolam, fentanyl, rocuronium, etomidate; Maintain: sevoflurane	96
Wang H et al.	2015	China	41 /31	73.5 ± 2.8	Lung cancer operation	I~II	①②③④⑤⑥	Induction: unified rapid induction; Maintain: propofol	36	Induction: unified rapid induction; Maintain: sevoflurane	36
Wang F et al.	2017	China	32 /18	72.5 ± 3.0	Lung cancer operation	N	①②③④⑤⑥	Induction: midazolam, fentanyl, rocuronium, etomidate; Maintain: propofol	50	Induction: midazolam, fentanyl, rocuronium, etomidate; Maintain: sevoflurane	50
Zhao et al.	2014	China	80 /30	73.5 ± 2.0	Lung cancer operation	I~II	①②③④⑤⑥	Induction: fentanyl and vecuronium bromide; Maintain: propofol 2~4 mg/kg/min	50	Induction: fentanyl and vecuronium bromide; Maintain: sevoflurane	60
Chen et al.	2015	China	43 /35	69.2 ± 3.2	Lung cancer operation	N	①②③④⑤⑥	Induction: midazolam, propofol, fentanyl and vecuronium bromide; Maintain: propofol 6~10 mg/kg/min	39	Induction: midazolam, propofol, fentanyl and vecuronium bromide; Maintain: sevoflurane	39
Huang et al.	2015	China	50 /40	68.2 ± 1.3	Lung cancer operation	N	①②③④⑤⑥	Induction: rocuronium, fentanyl, midazolam, etomidate; Maintain: propofol	45	Induction: rocuronium, fentanyl, midazolam, etomidate; Maintain: sevoflurane	45
Lin et al.	2017	China	54/40	68.23 ± 1.32	Lung cancer operation	I~II	①②③④⑥	Induction: propofol, midazolam, vecuronium, fentanyl; Maintain: propofol	40	Induction: propofol, midazolam, vecuronium, fentanyl; Maintain: sevoflurane	54
Zhang et al.	2017	China	41/29	P: 74.8 ± 2.1; S: 74.3 ± 2.5	Lung cancer operation	I~II	②③④	Induction: fentanyl, etomidate, midazolam, rocuronium; Maintain: propofol	35	Induction: fentanyl, etomidate, midazolam, rocuronium; Maintain: sevoflurane	35
Yang et al.	2017	China	84/36	71.9 ± 2.5	Lung cancer operation	N	①②③④⑤⑥	Induction: unified rapid induction; Maintain: propofol	60	Induction: unified rapid induction; Maintain: sevoflurane	60
Tian et al.	2017	China	38/24	P: 68.3 ± 13.5; S: 65.5 ± 16.2	Lung cancer operation	I~II	①③⑥	Induction: midazolam, fentanyl, propofol; Maintain: propofol	31	Induction: midazolam, fentanyl, sevoflurane; Maintain: propofol	31

*N* Not mentioned, *ASA* American society of anesthesiology, ① = Preoperative MMSE score, ② = MMSE score at 6 h after surgery, ③ = MMSE score at 1 day after surgery, ④ = MMSE score at 3 day after surgery, ⑤ = MMSE score at 7 day after surgery, ⑥ = Plasma S100β protein level at 1 day after surgery, *P* Propofol, *S* Sevoflurane

Quality Evaluation: The Cochrane System Evaluation Criteria was used for this evaluation. We used the Cochrane Risk Bias Assessment Tool to analyze the literature bias [11].

### Patient involvement

There was no patient involvement in the design and implementation of this study.

### Statistical analysis

The primary endpoints are postoperative mini-mental state examination (MMSE) scores at various time points; the secondary endpoint is serum S100beta concentration 24 h after surgery. STATA 13.0 (StataCorp LP, College Station, TX, USA) software was used for statistical analysis. In order to eliminate the influence of different units and differences in the means among different research studies, we analyzed the standard mean difference (SMD) and its 95% CI. We used the Galbr plot, I^2^ test and Cochran’s Q-test to determine whether the results are heterogeneous, and at the same time, we analyzed the heterogeneity by calculating I^2^. If the *P* value> 0.1 and I2 < 50%, the heterogeneity between the results is not apparent, so that a fixed effect model would be used for this meta-analysis; when *P* ≤ 0.1 and I2 ≥ 50%, it indicates that the results of the studies are heterogeneous. If the heterogeneity is apparent, then it could be eliminated by searching for the source of heterogeneity and analyzing the sensitivity; if the source of heterogeneity is not bright, the meta-analysis would be performed using a random-effect model. A bias risk assessment tool was used to assess the risk of bias. Detailed explanations of the mentioned analyses were included in Table 3. Two-tailed *P* values less than 0.05 were considered as statistically significant.

**Table 3:** The statistical methods used in this meta-analysis and their explanation

Goals and Usages	Statistic Methods	Explanations and Instructions
To evaluate heterogeneity between the included studies	Galbr plot	In Galbr figure, if the points all fall within the area between the upper line and the lower line, it can be taken as an evidence of homogeneity; otherwise, there is heterogeneity.
	Cochran’s Q test	Cochran’s Q test is an extension to the McNemar test for related samples that provides a method for testing for differences between three or more matched sets of frequencies or proportions. Heterogeneity was also considered significant if *P* < 0.05 using the Cochran’s Q test.
	I^2^ index test	The I^2^ index measures the extent of true heterogeneity dividing the difference between the result of the Q test and its degrees of freedom (k – 1) by the Q value itself, and multiplied by 100. I^2^ values of 25, 50 and 75% were used as evidence of low, moderate and high heterogeneity, respectively.
To examine the stability of the pooled results	Sensitivity analysis	A sensitivity analysis was performed using the one-at-a-time method, which involved omitting one study at a time and repeating the meta-analysis. If the omission of one study significantly changed the result, it implied that the result was sensitive to the studies included.
Publication bias test	Contour-enhanced funnel plot	Visual inspection of the Contour-enhanced funnel plots was used to assess potential publication bias. Asymmetry in the plots, which may be due to studies missing on the left-hand side of the plot that represents low statistical significance, suggested publication bias. If studies were missing in the high statistical significance areas (on the right-hand side of the plot), the funnel asymmetry was not considered to be due to publication bias

## Results

### Search results

**Fig. 1:**
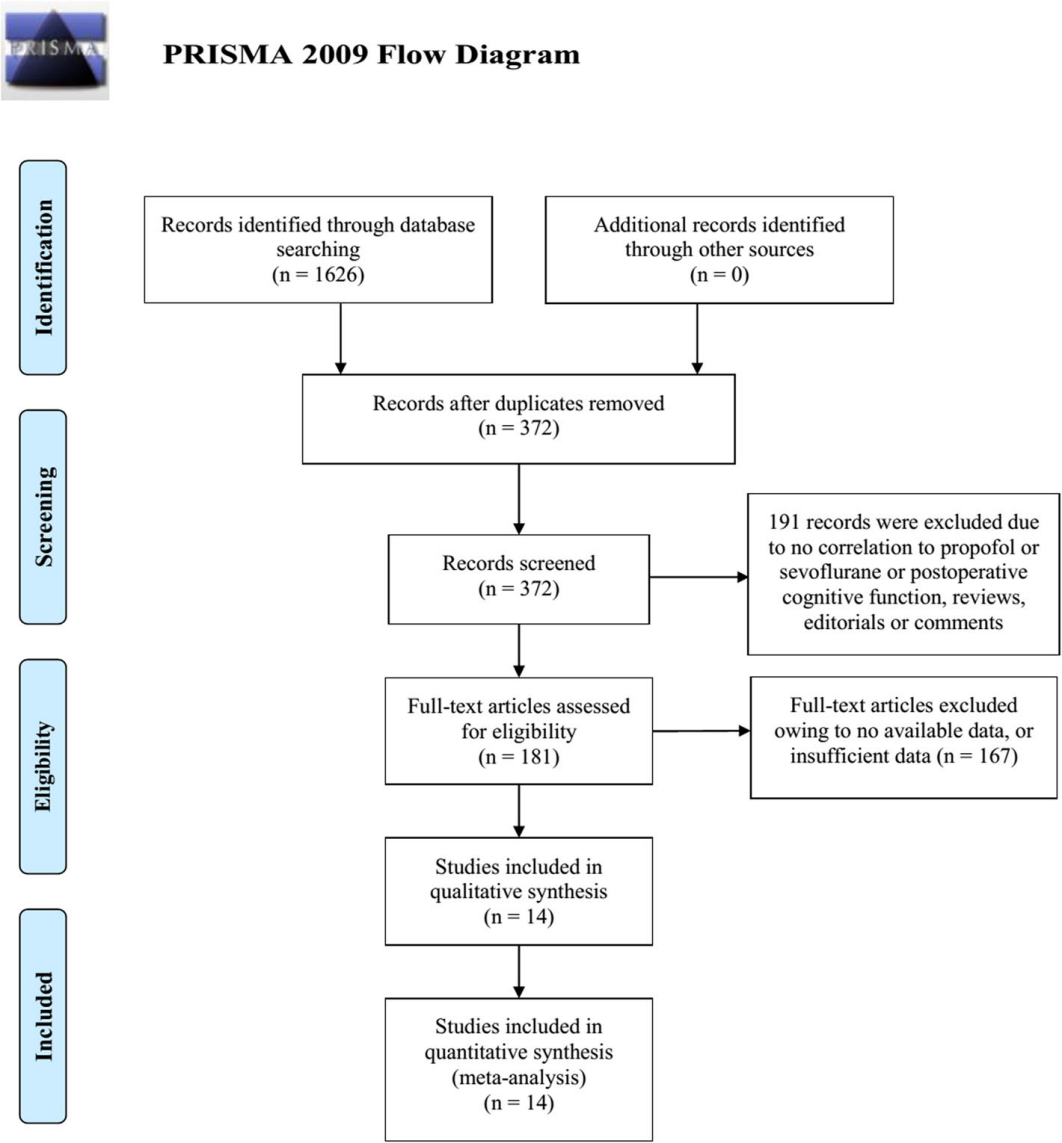
Literature search and selection of articles

The flowchart summarizing the study selection process following the PRISMA statement was reported in Fig. 1. A total of 1626 studies were identified in the initial search, including 108 studies from PubMed, 1131 studies from EMBASE, 148 studies from Cochrane Library, 195 studies from Web of Science, 44 studies from CNKI, and one academic meeting abstracts (Table 4). After screening based on inclusion and exclusion criteria, 372 articles were retrieved as eligible and then reviewed by two independent reviewers. Finally, fourteen studies, including 1404 patients, were included in the final meta-analysis [12–25].

**Table 4:** Searching strategies and results for different databases (cut-off date: April 20, 2017)

Database	Database URL	Search strategy		Results
Pubmed	https://www.ncbi.nlm.nih.gov/pubmed/	(“sevoflurane” [Supplementary Concept] OR “sevoflurane” [All Fields]) AND (“propofol” [MeSH Terms] OR “propofol” [All Fields]) AND (“lung” [MeSH Terms] OR “lung” [All Fields])		108
Embase	https://www.embase.com/	(‘sevoflurane’/exp. OR sevoflurane) AND (‘propofol’/exp. OR propofol) AND (‘lung’/exp. OR lung)		1131
Cochrane Library	http://www.cochranelibrary.com/	Sevoflurane AND Propofol AND lung:ti, ab, kw		148
Web of Science	http://apps.webofknowledge.com/	TOPIC: (Sevoflurane AND Propofol AND postoperative AND pain); Timespan: All years. Indexes: SCI-EXPANDED, SSCI, A&HCI, ESCI.		195
CNKI	http://www.cnki.net/	Search conditions: (topic = sevoflurane propofol lung cancer) (fuzzy matching), album navigation: all; database: literature cross-database search; search method: cross-database search Database: Literature		44
Searching results and information of relevant academic meeting abstracts				
Year	City	Meeting name	Article title	Whether included
2015	Beijing, P.R. China	Chinese seminar on translational medicine and integrative medicine	Difference of postoperative cognitive functions under propofol or sevoflurane anesthesia for lung cancer surgery	No

### Patient characteristics

In terms of patient race group, all studies were performed in patients of Asian backgrounds. There was one study published in English and 13 studies in Chinese. The characteristics of the studies included in this meta-analysis were listed in Table 2 in detail.

### Meta-analysis results and bias assessment results

**Fig. 2:**
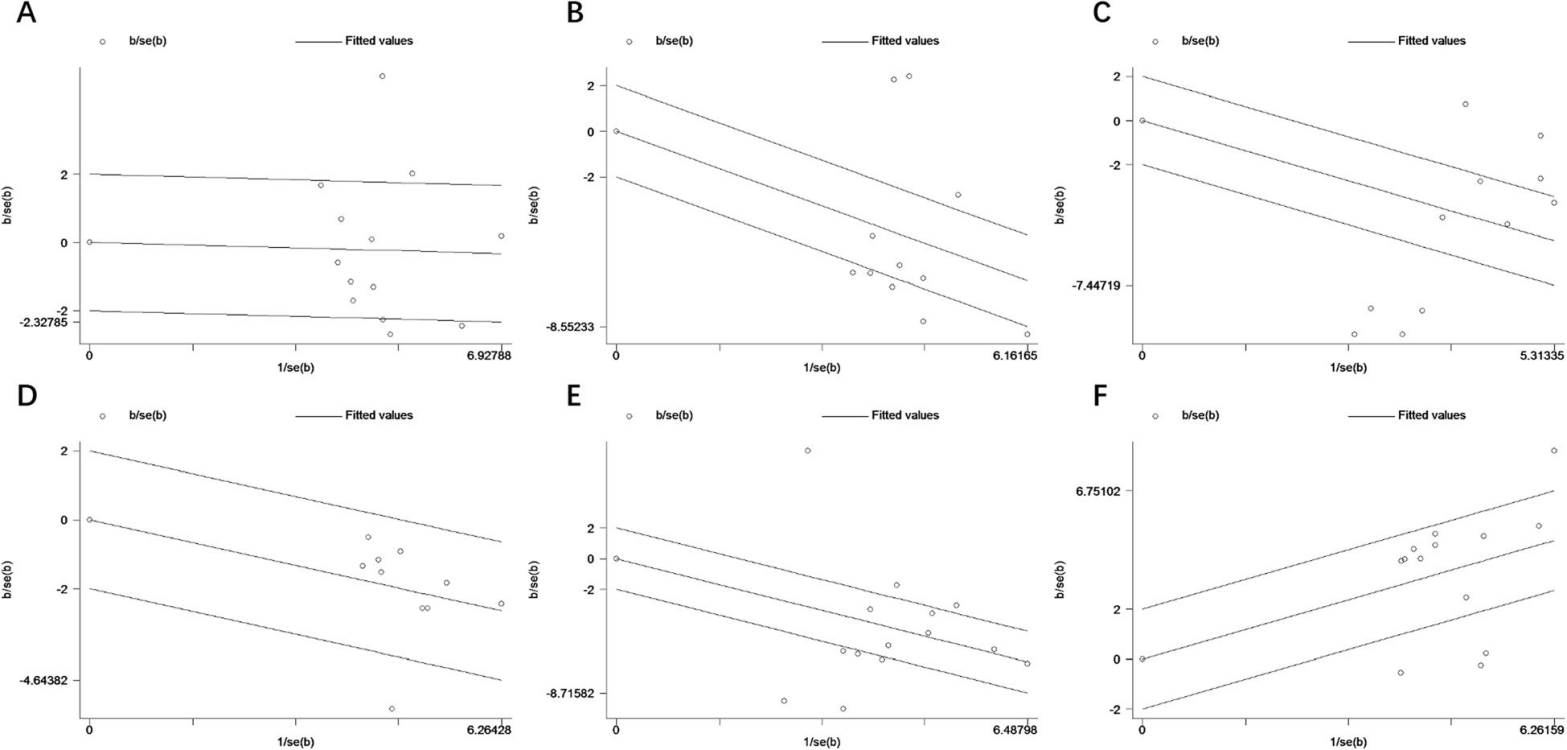
Galbr plots of the included studies focusing on the effects of propofol and sevoflurane on postoperative cognitive function (**a** preparative MMSE score; **b** MMSE score 6 h after surgery; **c** MMSE score 24 h after surgery; **d** MMSE score 3 days after surgery; **e** MMSE score 7 days after surgery; **f** serum S100beta concentration24 hours after surgery)

**Fig. 3:**
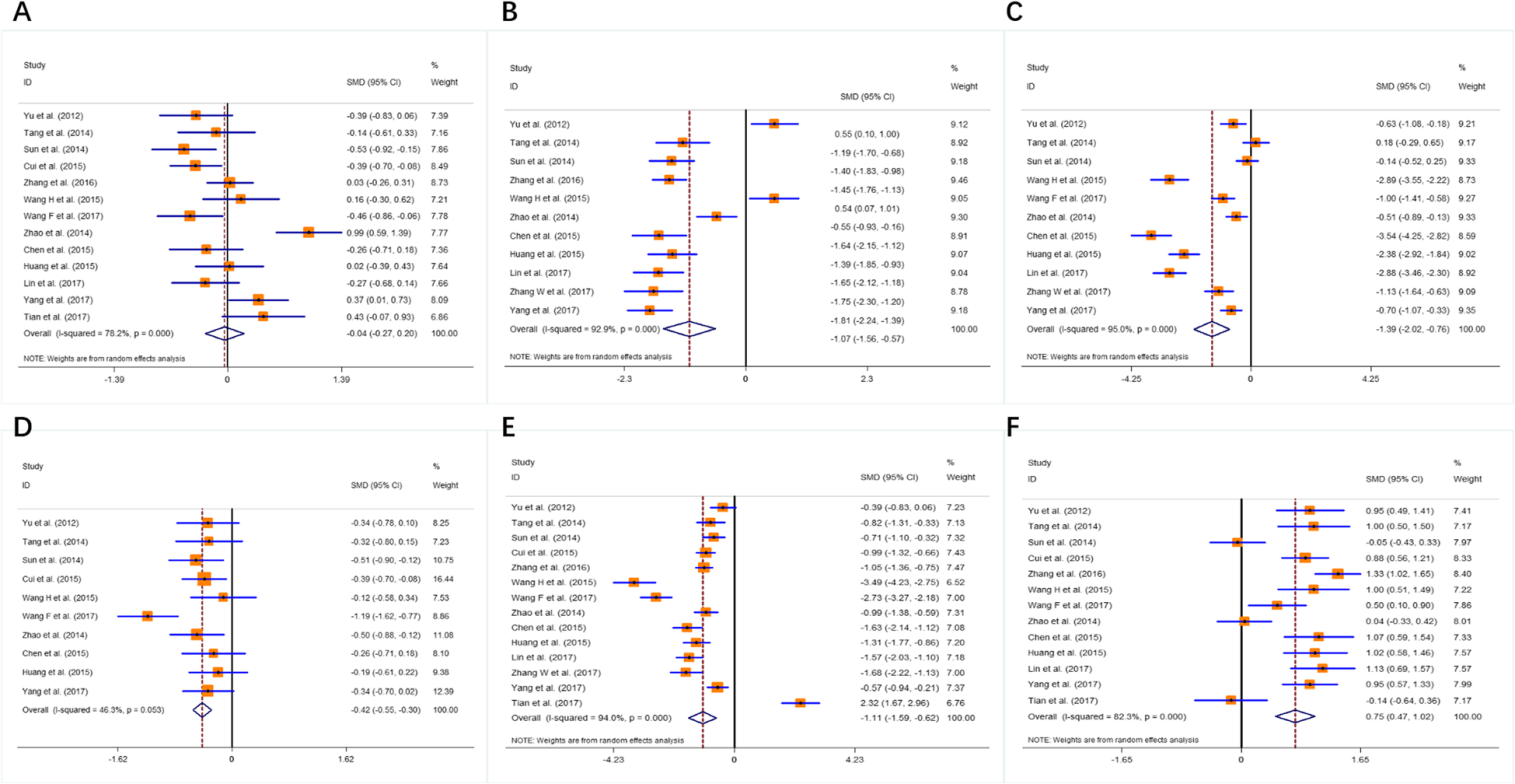
Forest plots of the included studies focusing on the effects of propofol and sevoflurane on postoperative cognitive function (**a** preparative MMSE score; **b** MMSE score 6 h after surgery; **c** MMSE score 24 h after surgery; **d** MMSE score 3 days after surgery; **e** MMSE score 7 days after surgery; **f** serum S100beta concentration 24 h after surgery)

The main results, including heterogeneity tests, effect models adopted accordingly, and the pooled SMDs with their 95% CI and the *P* value of this meta-analysis were presented in Table 5. The Galbr plots for the association between the use of narcotic drugs and postoperative cognitive function were shown in Fig. 2, suggesting that there was no heterogeneity only among the 10 studies [12, 13, 15, 17, 19–22, 24, 25] with continuous data focusing on MMSE scores 7 days after the surgery, but not among other comparisons. Using fixed-effects model, the pooled SMD for the 10 studies was − 0.422 (95% CI: − 0.549, − 0.295, Z = 6.52; *P* < 0.001), the pooled WMD was − 0.371 (95% CI: − 0.493, − 0.249, P < 0.001), indicating that in terms of MMSE scores 7 days postoperatively, propofol has a greater adverse effect on cognitive function in the elderly patients with lung cancer than sevoflurane. The pooled SMD or WMD in issues of preoperative MMSE scores suggested no statistical difference (SMD -0.038, 95% CI: − 0.274, 0.198; WMD − 0.040, 95% CI: − 0.288, 0.208; Z = 0.31; *P* = 0.753). Then the pooled SMD in issues of postoperative MMSE scores at different time points were calculated using the random-effects model (except the MMSE score-7d). There were significant differences in issues of MMSE 6 h (11 studies; SMD -1.391, 95% CI -2.024, − 0.757; WMD -1.922, 95% CI -2.571, − 1.274; *p* < 0.001), MMSE 24 h (14 studies; SMD -1.106, 95% CI -1.588, − 0.624; WMD -1.504, 95% CI -2.253, − 0.755; p < 0.001), MMSE 3d (11 studies; SMD -1.065, 95% CI -1.564, − 0.566; WMD -1.376, 95% CI -2.044, − 0.708; p < 0.001), MMSE 7d (10 studies; SMD -0.422, 95% CI -0.549, − 0.295; WMD -0.371, 95% CI -0.493, − 0.249; p < 0.001), and the serum S100beta concentration at 1 day after surgery (13 studies; SMD 0.746, 95% CI 0.475, 1.017; WMD 0.018, 95% CI 0.016, 0.020; *p* < 0.001) (Fig. 3). We assessed the risk of bias using the Cochrane risk of bias tool [11]. Table 6 reported detailed results from the risk of a bias assessment tool.

**Table 5:** The results of the meta-analysis for the effect of propofol and sevoflurane on postoperative cognitive function

Comparative items		Data type	Heterogeneity test							Test of Association							Publication bias
MMSE and protein marker	Items		Q value	d.f.	I-squared	Tau-squared	*P* Value	Heterogeneity	Effect model	Pooled WMD	WMD 95% CI	Pooled SMD	SMD 95% CI	Z value	*P* value	Statistical significance	
Preoperative cognitive function	Preoperative MMSE	Continuous	55.09	12	78.2%	0.1449	0.000	Yes	Random	-0.040	[−0.288, 0.208]	− 0.038	[− 0.274, 0.198]	0.31	0.753	No	No
Postoperative cognitive function evaluation	MMSE 6 h	Continuous	199.17	10	95.0%	1.0826	0.000	Yes	Random	−1.922	[−2.571, − 1.274]	−1.391	[− 2.024, −0.757]	4.30	0.000	Yes	No
	MMSE 24 h	Continuous	216.54	13	94.0%	0.7859	0.000	Yes	Random	−1.504	[−2.253, −0.755]	− 1.106	[− 1.588, − 0.624]	4.50	0.000	Yes	No
	MMSE 3d	Continuous	140.36	10	92.9%	0.6585	0.000	Yes	Random	−1.376	[−2.044, −0.708]	− 1.065	[− 1.564, − 0.566]	4.19	0.000	Yes	No
	MMSE 7d	Continuous	16.75	9	46.3%	NA	0.053	No	fixed	−0.371	[−0.493, − 0.249]	−0.422	[− 0.549, − 0.295]	6.52	0.000	Yes	No
Protein marker S100β	S100β 1d	Continuous	67.94	12	82.3%	0.2024	0.000	Yes	Random	0.018	[0.016, 0.020]	0.746	[0.475, 1.017]	5.39	0.000	Yes	No

**Table 6:** Study quality: review authors’ judgments about each risk of bias item for each included study

Author	Year	Random sequence generation	Allocation concealment	Blinding of participants and personnel	Blinding of outcome assessment	Incomplete outcome data	Selective reporting	Other sources of bias
Yu et al.	2012	Unclear risk	Unclear risk	Unclear risk	Unclear risk	Low risk	Unclear risk	Unclear risk
Tang et al.	2014	Unclear risk	Unclear risk	Unclear risk	Unclear risk	Low risk	Unclear risk	Unclear risk
Sun et al.	2014	Unclear risk	Unclear risk	Unclear risk	Unclear risk	Low risk	Unclear risk	Unclear risk
Cui et al.	2015	Unclear risk	Unclear risk	Unclear risk	Unclear risk	Low risk	Unclear risk	Unclear risk
Zhang et al.	2016	Unclear risk	Unclear risk	Unclear risk	Unclear risk	Low risk	Unclear risk	Unclear risk
Wang H et al.	2015	Unclear risk	Unclear risk	Unclear risk	Unclear risk	Low risk	Unclear risk	Unclear risk
Wang F et al.	2017	Random number table	Unclear risk	Unclear risk	Unclear risk	Low risk	Unclear risk	Unclear risk
Zhao et al.	2014	Unclear risk	Unclear risk	Unclear risk	Unclear risk	Low risk	Unclear risk	Unclear risk
Chen et al.	2015	Unclear risk	Unclear risk	Unclear risk	Unclear risk	Low risk	Low risk	Unclear risk
Huang et al.	2015	Random number table	Unclear risk	Unclear risk	Unclear risk	Low risk	Unclear risk	Unclear risk
Lin et al.	2017	Unclear risk	Unclear risk	Unclear risk	Unclear risk	Low risk	Unclear risk	Unclear risk
Zhang et al.	2017	Unclear risk	Unclear risk	Unclear risk	Unclear risk	Low risk	Unclear risk	Unclear risk
Yang et al.	2017	Low risk	Unclear risk	Unclear risk	Unclear risk	Low risk	Low risk	Unclear risk
Tian et al.	2017	Low risk	Unclear risk	Unclear risk	Unclear risk	Low risk	Low risk	Unclear risk

### Sensitivity analysis

**Fig. 4:**
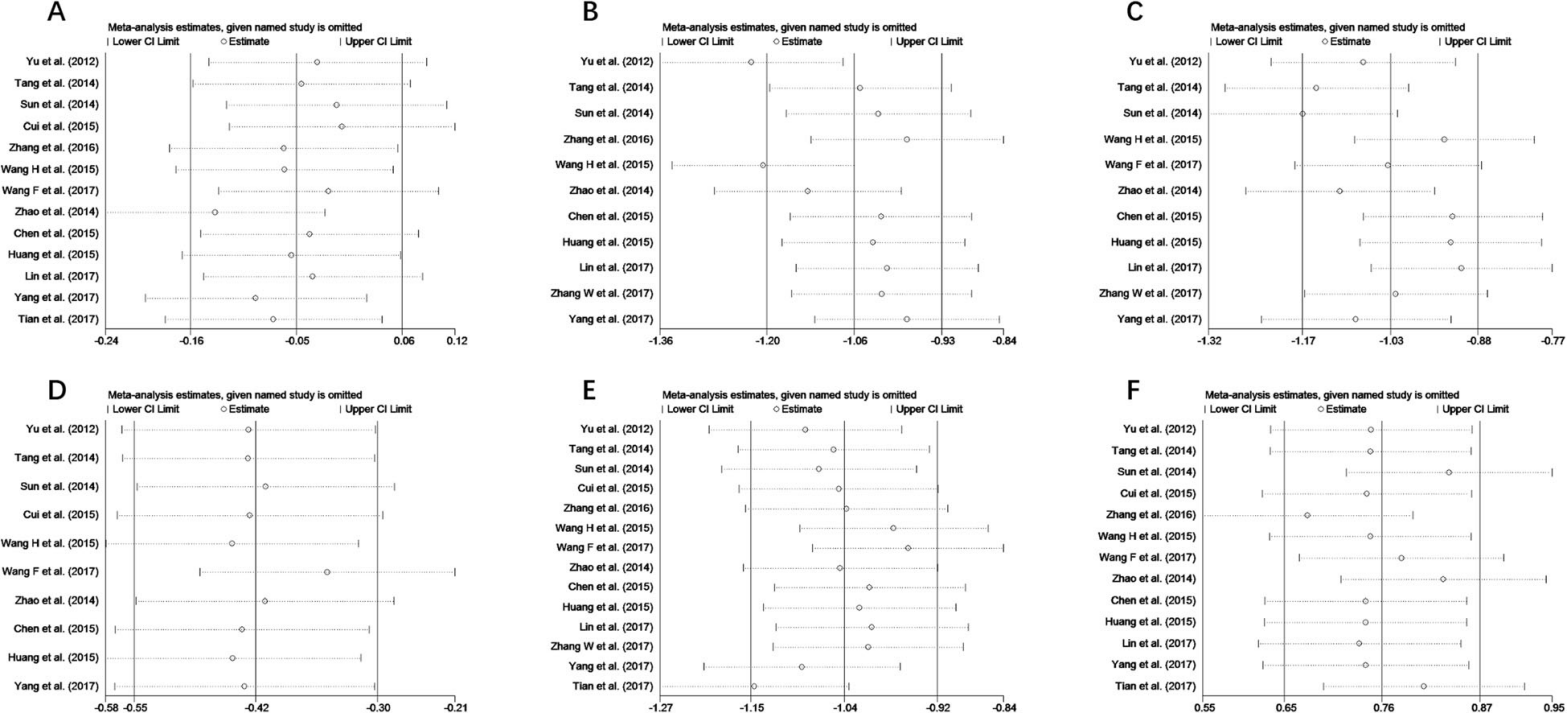
Sensitivity analyses of the included studies focusing on the effects of propofol and sevoflurane on postoperative cognitive function (**a** preparative MMSE score; **b** MMSE score 6 h after surgery; **c** MMSE score 24 h after surgery; **d** MMSE score 3 days after surgery; **e** MMSE score 7 days after surgery; **f** serum S100beta concentration 24 h after surgery)

To assess if a single study could affect the final SMDs, each study was removed one time, and the data re-pooled. The analysis results demonstrated that the pooled SMDs were not affected by deleting every single study. Figure 4 showed sensitivity analysis results in issues of postoperative pain and propofol/remifentanil use.

### Publication bias

**Fig. 5:**
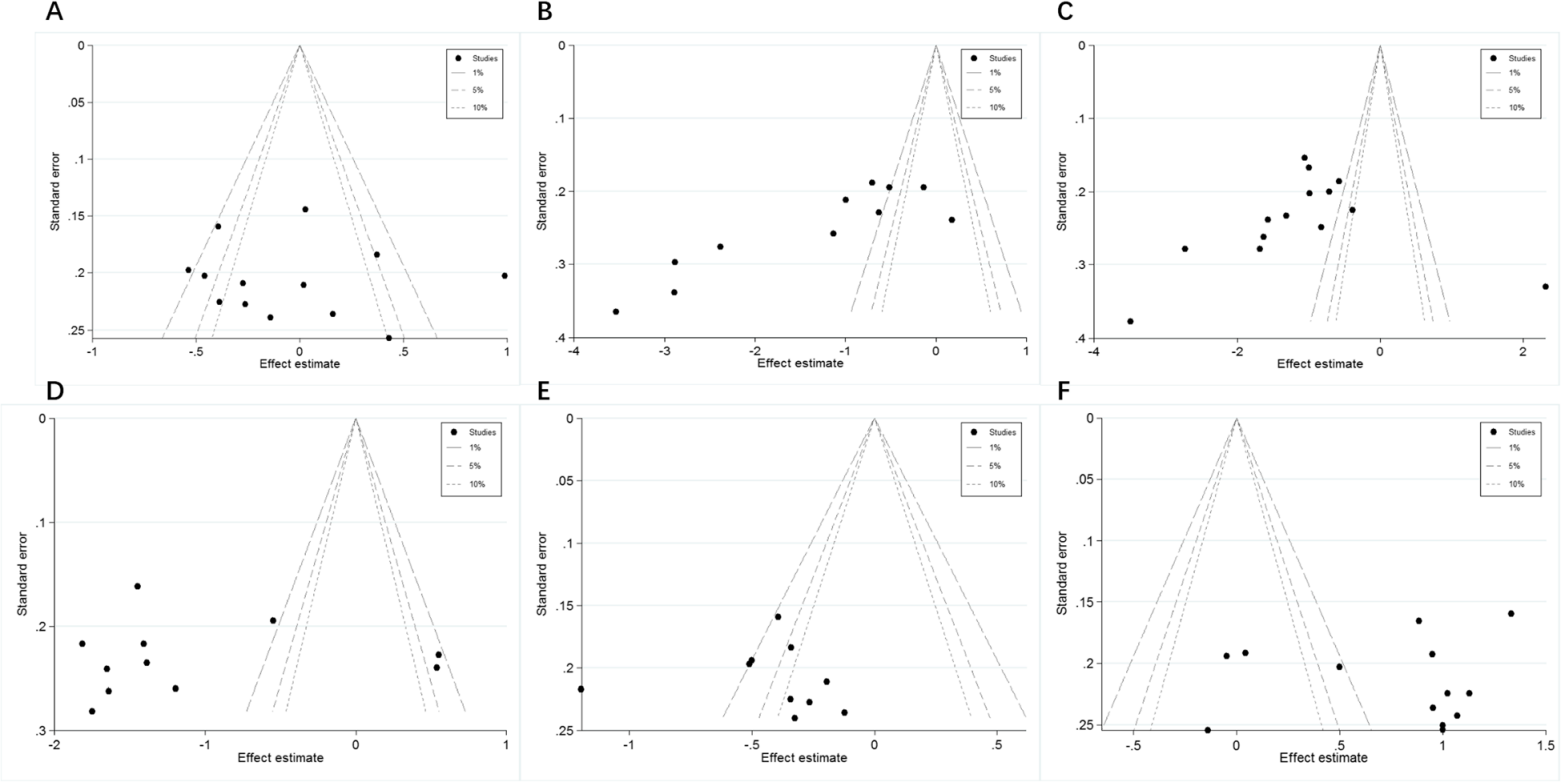
Contour-enhanced funnel plots of the included studies focusing on the effects of propofol and sevoflurane on postoperative cognitive function (**a** preparative MMSE score; **b** MMSE score 6 h after surgery; **c** MMSE score 24 h after surgery; **d** MMSE score 3 days after surgery; **e** MMSE score 7 days after surgery; **f** serum S100beta concentration 24 h after surgery)

The contour-enhanced funnel plots (this term’s explanation could be seen in Table 3) were adopted to estimate potential publication biases, showing that most of the studies had missing areas for low statistical significance (the left-hand side of the plot), indicating no publication bias in present studies (Fig. 5).

## Discussion

The potential risk for cognitive impairment following surgery and anesthesia is a common concern, especially in the elderly and more fragile patients. The risk for various neurocognitive effects is thus an area of importance. The independent impact of surgery and anesthesia is still not known. Likewise, the independent effect of different drugs used during anesthesia is a matter of debate, as is the number and amounts of drugs used and the “depth of anesthesia.” So, understanding the drug-related phenomenon and mechanisms for postoperative cognitive impairment is essential. This meta-analysis aims to compare the effects of propofol and sevoflurane anesthesia on postoperative cognitive function in elderly patients with lung cancer. This meta-analysis compared the effects of propofol and sevoflurane anesthesia on postoperative cognitive function in elderly patients (> 60-year-old) with lung cancer. Results suggested that propofol has a more significant adverse effect on cognitive function in elderly patients with lung cancer than sevoflurane. There were significant differences in issues of MMSE 6 h, MMSE 24 h, MMSE 3d, MMSE 7d, and the serum S100beta concentration at 1 day after surgery (all *p* < 0.01). Regarding the two drugs analyzed, the half-life of elimination of propofol has been estimated to be between 2 and 24 h. However, its duration of clinical effect is much shorter, because propofol is rapidly distributed into peripheral tissues. When used for IV sedation, a single dose of propofol typically wears off within minutes. The half-life of elimination of sevoflurane is 15–23 h.

At present, there is no uniform standard for the assessment of cognitive dysfunction in the world, and the most commonly used standard clinically is the MMSE score [26]. Studies found that the sensitivity and specificity of the MMSE method for assessing cognitive brain function were 87 and 82%, respectively [27]. The MMSE method is feasible and straightforward and is widely used for the screening of clinical cognitive dysfunction and cognitive decline [28]. The results of this meta-analysis showed that the MMSE scores at 6 h, 1d, 3d, and 7d after intravenous propofol anesthesia were significantly lower than those of sevoflurane. The reason may be that the sevoflurane has a shorter action time and is eliminated quickly. Laboratory data also showed that rats pretreated with high concentrations of sevoflurane could be effectively protected from focal cerebral ischemia, thus reducing neurological deficit scores, the volume of cerebral infarction, and cerebral edema areas. The incidence of post-cognitive disorders is adversely associated with higher concentrations of sevoflurane as a potential protective factor in non-cardiovascular procedures [29]. Experimental data have demonstrated that it may be because of the up-regulation of the expression levels of NR1 and NR2 subunits of hippocampal N-methyl-D-aspartate receptors that sevoflurane has a slighter effect on cognitive function in the elderly patients than propofol [30].

On a contour-enhanced funnel plot, contours of statistical significance are overlaid on the funnel plot. Adding contours of statistical significance facilitates the assessment of whether the areas where studies exist are areas of statistical significance and whether the areas where studies are potentially missing correspond to areas of low statistical significance. Generally, if studies appear to be missing in areas of low statistical significance, then it is possible that the asymmetry is due to publication bias. Conversely, studies perceived to be missing areas of high statistical significance likely do not suffer from publication bias as a source of funnel asymmetry. In the present meta-analysis, the funnel plot indicated no publication bias.

This meta-analysis has a few limitations. Firstly, we included only 14 articles. Although all these studies were conducted with propofol anesthesia and sevoflurane anesthesia as the experimental group and the control group, respectively, the doses of the drugs used for the elderly patients with lung cancer, the length of the operation time, and the use of adjuvant drugs were not the same. So, the problem of generalizability might exist. Secondly, most literature tends to report positive outcomes, while the studies with negative results are often not reported. Though not suggested via contour-enhanced funnel plots, the possibility of potential publication bias in included studies should not be overlooked. Thirdly, we did not perform a grey literature search, which might cause overestimations of effect sizes. Grey literature stands for manifold document types produced on all levels of government, academics, business and industry in print and electronic formats that are protected by intellectual property rights, of sufficient quality to be collected and preserved by libraries and institutional repositories, but not controlled by commercial publishers; i.e. where publishing is not the primary activity of the producing body. Fourthly, there are several tools more extensive tools available for assessment of recovery; however, in the present study, we only adopted MMSE as the outcome because of the data availability (MMSE is the most commonly used for screening). Although one may argue that merely presenting repeated MMSE scores is not sufficient for assessment of cognitive capacity and discriminating differences between groups, we believe that at this present time, due to the data availability mentioned above, the MMSE could say something. Fifthly, most of the included studies did not report enough the details of each patient, so it is not easy to separate between emergence reaction within hours from emergence, early cognitive changes - delirium and more protracted changes. Additionally, the many included studies had a relatively low methodology quality, so more rigorous large-scale randomized controlled trials are needed. Sixthly, the vast majority of the studies were conducted in China. The inter-racial variability of propofol anesthesia is well described. Therefore, it is not sure if the results are valid for other races. Seventhly, as is understood, co-administered drugs differed in included studies, but did any of the studies include regional anesthetic techniques? Could the greater peri-operative hypotension sometimes see with propofol have been a cause? Due to lacking these related data, the accuracy of the study’s conclusions will be questioned.

## Conclusions

In summary, propofol has a more significant adverse effect on postoperative cognitive function in lung cancer patients than sevoflurane. In the included studies, some of the documents are of low quality and may affect the stability and reliability of the final results. Therefore, larger samples, more rigorous design, and higher quality tests are still needed for verification.

## Data Availability

All data has been included in the manuscript.

## Abbreviations

ASA: America Society of Anesthesiologist
CI: Confidence interval
CNKI: China National Knowledge Infrastructure
CNS: Central nervous system
MMSE: Mini-mental state examination
SMD: Standard mean difference
